# Optimal structure of metaplasticity for adaptive learning

**DOI:** 10.1371/journal.pcbi.1005630

**Published:** 2017-06-28

**Authors:** Peyman Khorsand, Alireza Soltani

**Affiliations:** Department of Psychological and Brain Sciences, Dartmouth College, New Hampshire, United States of America; New York University, UNITED STATES

## Abstract

Learning from reward feedback in a changing environment requires a high degree of adaptability, yet the precise estimation of reward information demands slow updates. In the framework of estimating reward probability, here we investigated how this tradeoff between adaptability and precision can be mitigated via metaplasticity, i.e. synaptic changes that do not always alter synaptic efficacy. Using the mean-field and Monte Carlo simulations we identified ‘superior’ metaplastic models that can substantially overcome the adaptability-precision tradeoff. These models can achieve both adaptability and precision by forming two separate sets of meta-states: reservoirs and buffers. Synapses in reservoir meta-states do not change their efficacy upon reward feedback, whereas those in buffer meta-states can change their efficacy. Rapid changes in efficacy are limited to synapses occupying buffers, creating a bottleneck that reduces noise without significantly decreasing adaptability. In contrast, more-populated reservoirs can generate a strong signal without manifesting any observable plasticity. By comparing the behavior of our model and a few competing models during a dynamic probability estimation task, we found that superior metaplastic models perform close to optimally for a wider range of model parameters. Finally, we found that metaplastic models are robust to changes in model parameters and that metaplastic transitions are crucial for adaptive learning since replacing them with graded plastic transitions (transitions that change synaptic efficacy) reduces the ability to overcome the adaptability-precision tradeoff. Overall, our results suggest that ubiquitous unreliability of synaptic changes evinces metaplasticity that can provide a robust mechanism for mitigating the tradeoff between adaptability and precision and thus adaptive learning.

## Author summary

Successful learning from our experience and feedback from the environment requires that the reward value assigned to a given option or action to be updated by a precise amount after each feedback. In the standard model for reward-based learning known as reinforcement learning, the learning rates determine the strength of such update. A large learning rate allows fast update of values (large adaptability) but introduces noise (small precision), whereas a small learning rate does the opposite. Thus, learning seems to be bounded by a tradeoff between adaptability and precision. Here, we asked whether there are synaptic mechanisms that are capable of adjusting the brain’s level of plasticity according to reward statistics, and, therefore, allow the learning process to be adaptive. We showed that metaplasticity, changes in the synaptic state that shape future synaptic modifications without any observable changes in the strength of synapses, could provide such a mechanism and furthermore, identified the optimal structure of such metaplasticity. We propose that metaplasticity, which sometimes causes no observable changes in behavior and thus could be perceived as a lack of learning, can provide a robust mechanism for adaptive learning.

## Introduction

To successfully learn from reward feedback, the brain must adjust how it responds to and integrates reward outcomes, since reward contingencies can unpredictably change over time [1,2]. At the heart of this learning problem is a tradeoff between adaptability and precision. On the one hand, the brain must rapidly update reward values in response to changes in the environment; on the other hand, in the absence of any such changes, it must obtain accurate estimates of those values. This tradeoff, which we refer to as the adaptability—precision tradeoff [3,4], can be easily demonstrated in the framework of reinforcement learning [5]. According to this framework, larger learning rates result in higher adaptability but lower precision, and smaller learning rates give rise to lower adaptability but higher precision. In recent years, the failure of conventional reinforcement learning (RL) models to capture the level of adaptability and precision demonstrated by humans and animals has led to alternative explanations for how we deal with uncertainty and volatility in the environment [1,6,7,8]. However, most of these solutions for adjusting learning require complicated calculations, and their underlying neural substrates are unknown.

Given the central role of synapses in learning, we asked whether there are local synaptic mechanisms that can adjust the level of plasticity according to reward statistics and, therefore, allow the learning process to be adaptable. A candidate mechanism for such adjustment is metaplasticity, defined as changes in the synaptic state that shape the direction, magnitude, and duration of future synaptic changes without any observable change in the efficacy of synaptic transmission [9,10,11,12]. Extending our recent heuristic model of reward-dependent metaplasticity, which enables adjustment of learning to reward uncertainty [3], we examined a general class of metaplastic models to identify features that are beneficial for mitigating the adaptability-precision tradeoff (APT) during the estimation of the probability of binary reward.

Using the mean-field and Monte Carlo simulations, we identified optimal metaplastic models that can substantially overcome the APT. These models, which we refer to as ‘superior’ models, achieve both adaptability and precision by forming two separate sets of meta-states: reservoirs and buffers. Synapses in reservoir meta-states do not change their efficacy upon reward feedback, whereas those in buffer meta-states can change their efficacy. In superior models, rapid changes in efficacy are limited to synapses occupying buffers, creating a bottleneck that reduces noise without significantly decreasing adaptability. In contrast, more-populated reservoirs can generate a strong signal without manifesting any observable plasticity. Comparison of the behavior of our model and a few competing models during a dynamic probability estimation task revealed that superior metaplastic models perform close to optimally for a wider range of model parameters. However, superior models were suboptimal when precision was defined in the absolute term (absolute value of the difference between estimated and actual probabilities) rather than relative (the ability to distinguish neighboring values of probability), indicating that the brain could use different objectives to deal with reward uncertainty. Finally, we showed that metaplasticity provides a robust mechanism for mitigating the APT, and that metaplastic transitions are crucial, since replacing these transitions with plastic ones reduces the ability of the model to mitigate the APT. Altogether, our results illustrate how metaplasticity can mitigate one of the most fundamental tradeoffs in learning and, moreover, reveal the critical features of metaplasticity that contribute to adaptive learning.

## Results

### The adaptability-precision tradeoff

To study the relationship between adaptability and precision, we considered a general problem of estimating reward probability from a stream of binary outcomes (reward, no reward). We defined adaptability and precision in the context of this estimation task in order to quantify the adaptability-precision tradeoff (APT). We assumed that the estimation of reward probability is performed by a set of synapses and, thus, reward probability is stored in the strength of these synapses. As a result, adaptability in estimation of reward probability requires these synapses to change their strengths quickly whereas precision requires that the strength of synapses can discriminate between neighboring values of reward probability (see below).

**Fig 1:**
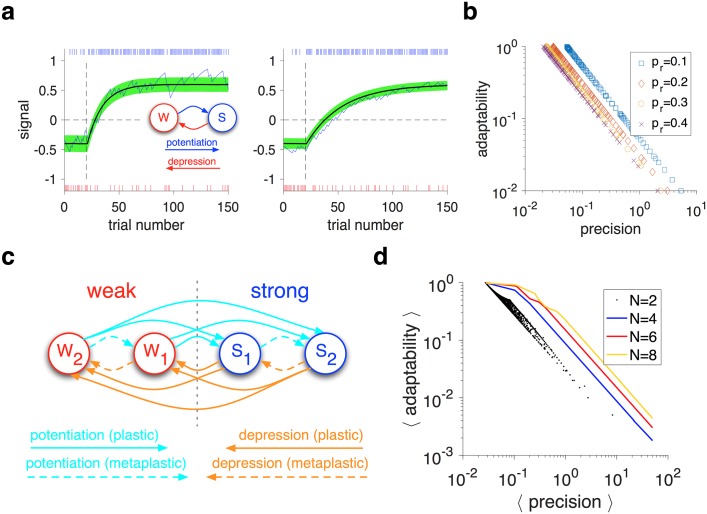
The adaptability-precision tradeoff in the plastic and metaplastic models. (**a**) The APT in estimating reward probability for the binary plastic model. Plotted is the signal in the binary plastic model in response to a sudden change in reward probability from 0.3 to 0.8 on trial 20. The blue curve shows an example instance of estimation in response to a reward sequence (tick marks on top and bottom indicate reward and no reward, respectively). The black curve shows the average over many instances and the green shade indicates the standard deviation over those instances. Decreasing the transition rates (from *t*^+^ = *t*^−^ = 0.07 in the left panel to *t*^+^ = *t*^−^ = 0.03 in the right) resulted in noise reduction in the asymptotic value of the signal, but at the expense of slower convergence to this asymptotic value. The inset shows the model with binary plastic synapses (W: weak; S: strong). (**b**) The APT manifests itself for different learning rates for different reward probabilities. Plotted is the adaptability, as a function of the precision for different values of *p*_*r*_. Each dot corresponds to a specific set of parameter values. The APT is stronger as *p*_*r*_ becomes closer to 0.5 (**c**) A schematic of a general model of metaplasticity with four ordered meta-states (*N* = 4). Synapses can transition between different meta-states with either weak or strong synaptic efficacies. (**d**) The APT in the binary plastic model (*N* = 2), and superior metaplastic models with different numbers of meta-states. Plotted is the average adaptability (over different values of *p*_*r*_) as a function of the average precision in different models. For the binary plastic model, each dot corresponds to a specific set of parameter values. For metaplastic models (*N* = 4,6,8), each outline connects models with optimized adaptability for a given value of average precision.

The estimation of reward probability can be preformed with a model consisting of synapses that follow a stochastic reward-dependent plasticity rule [13–15] (Fig 1a). In this model, which we refer to as the ‘plastic’ model, synapses are binary so they can be in the weak or strong state [16,17]. Weak synapses can be potentiated on rewarded trials with a probability *t*^+^ (potentiation rate), whereas strong synapses can be depressed on unrewarded trials with a probability *t*^−^ (depression rate). The difference between the fractions of synapses that are in the strong and weak states determines the signal stored in these synapses, reflecting the model’s estimate of reward probability. This model is equivalent to a simple RL model based on reward prediction error and can provide an unbiased estimate of reward probability when potentiation and depression rates are equal (S1 Text).

For a given value of reward probability, *p*_*r*_, the steady state of the model can be used to calculate the signal, and the weighted average change in signal due to single potentiation and depression events (‘one-step’ noise) provides a good proxy for noise (see Methods). We measured precision with the ability of the model to differentiate between adjacent reward probabilities instead of a more conventional definition based on the difference between the estimated and actual probabilities. We adopted the former definition because encoding and representation of reward information are inherently relative in the brain, since the probability estimated by a set of synapses can be easily scaled and biased by changes in the input neural firing to these synapses. Therefore, we defined the ‘precision’ (ℙ) as equal to the sensitivity of the model’s signal to changes in reward probabilities (‘sensitivity’), divided by noise in the signal. The ‘adaptability’ (A) of a model in estimating reward probability was defined as the rate at which the fractions of meta-states approach their final values (see Methods).

Because of the simplicity of the plastic and RL models, adaptability and precision can be analytically computed for these models (S2 Text). Both these models show a strict APT, since the product of adaptability and precision is independent of model parameters and only depends on reward probability (Fig 1a and 1b). Importantly, adopting different values for the potentiation and depression rates (or equivalently the learning rates in RL) cannot improve the APT. Rather, adoption of different values slightly alters the average values of adaptability and precision over a set of reward probabilities (Fig 1d and S1 Fig).

### Metaplasticity can mitigate the adaptability-precision tradeoff

Here, we considered a general model of metaplasticity and used optimization to identify the superior metaplastic models for mitigating the APT. Our general model of metaplasticity consisted of multiple meta-states associated with one of the two values of synaptic efficacy (weak and strong), and all possible transitions between these meta-states (Fig 1c; see Methods). In this model, the difference between the fractions of synapses that are in the strong and weak meta-states determines the signal (*S*) stored in these synapses, reflecting the model’s estimate of reward probability. Importantly, we assumed that metaplastic transitions have a consistent order, and thus, within the set of weak and strong meta-states, there are multiple meta-states with different levels of depth (Fig 1c). Since we were interested in conditions under which metaplasticity can improve the APT, we examined ‘superior’ metaplastic models (i.e. those which optimized A × ℙ for a given value of ℙ).

We found that for many model parameters, the APT can be mitigated by superior metaplastic models that consist of as few as four meta-states (Fig 1d; S2 Fig). These superior models overcame the APT by exhibiting three important characteristics: differential adjustments of learning based on reward probability; matching of sensitivity to noise; and optimal adaptability. Firstly, the learning on rewarded and unrewarded trials was differentially adjusted according to reward probability. Secondly, the sensitivity of the signal to reward probability matched the level of noise (sensitivity-to-noise matching), and this matching was improved with larger numbers of meta-states. Thirdly, the adaptability of the models was optimized for a given level of noise (see below).

**Fig 2:**
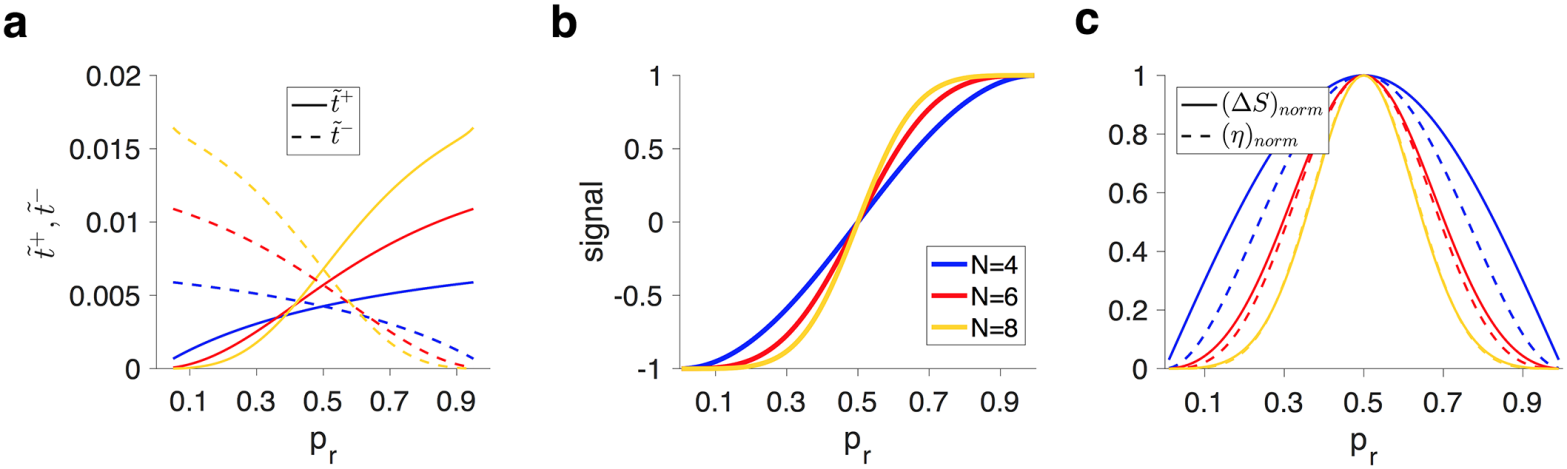
Adjustment of learning to reward probability in the superior metaplastic models. (**a**) Plotted are the effective learning rates for potentiation (${\tilde{t}}^{+}$) and depression (${\tilde{t}}^{-}$) events as a function of *p*_*r*_. The effective learning rate on potentiation (depression) events increases (decreases) as reward probability increases, with a crossover at *p*_*r*_ = 0.5. (**b**) Signal in superior metaplastic models. (**c**) Matching of the sensitivity to noise in the metaplastic models. Plotted are the normalized sensitivity (d*S*/d*p*_*r*_, denoted as (Δ*S*)_*norm*_) and one-step noise (*η*) as a function of *p*_*r*_ for three examples of superior metaplastic models with different numbers of meta-states. The sensitivity profile better matches the noise profile as *N* increases.

The first characteristic of superior models is that learning was naturally adjusted according to reward probability without any changes in the model’s parameters. To show this adjustment, we computed the ‘effective’ learning rates for potentiation and depression events for a given value of *p*_*r*_ (see Methods). The effective learning rate assigned a single rate to transitions between the weak and strong meta-states or vice versa (plastic transitions, Fig 1c), which are the only transitions that can change synaptic efficacy and thus the signal. We found that the effective learning rate on rewarded trials (${\tilde{t}}^{+}$) was close to zero for small values of *p*_*r*_ but monotonically increased as *p*_*r*_ increased (Fig 2a). At the same time, the effective learning rate on unrewarded trials (${\tilde{t}}^{-}$) was large when *p*_*r*_ was close to zero and decreased as *p*_*r*_ increased. The effective learning rates on rewarded and unrewarded trials crossed over at 0.5 due to the symmetry in models parameters with respect to reward and no reward.

To understand why these adjustments are beneficial for mitigating the APT, one should note that in the RL model, the convergence to the final estimate of reward probability (when *p*_*r*_ is small) slows down as more negative outcomes (no reward) are observed, since reward prediction error becomes smaller for unrewarded trials. This property limits adaptability. At the same time, the response to a positive outcome (reward) increases since reward prediction error increases on rewarded trials, and this property increases noise. In contrast, metaplastic models increase ${\tilde{t}}^{-}$ as the models receive more negative outcomes allowing them to slow their convergence to the final value of probability estimate to a lesser degree. On the other hand, decreasing ${\tilde{t}}^{+}$ makes the estimate more robust against sporadic positive outcomes (noise). The opposite happens when *p*_*r*_ becomes closer to one.

These complementary adjustments in learning resulted in a sigmoid-shape signal for superior metaplastic models (Fig 2b), which in turn, gives rise to the second characteristic of superior models, the match between the sensitivity and the noise level (Fig 2c). More specifically, the maximum sensitivity (d*S*/d*p*_*r*_) for superior models occurred at *p*_*r*_ = 0.5, such that the steepest part of the signal matched the maximum level of noise (Fig 2c). Additionally, for a given level of precision, the signal became a steeper function of reward probability and the maximum sensitivity increased as the number of meta-states increased (Fig 2b). Importantly, the slope of the signal (i.e. sensitivity) at *p*_*r*_ = 0.5 was linearly proportional to the ratio of effective learning rates around *p*_*r*_ = 0.5, indicative of a direct relationship between the sensitivity-to-noise matching and adjustment of learning to reward probability. The adjustments occur in metaplastic models without any changes in parameters; as reward probability deviates from 0.5 (say when *p*_*r*_ > 0.5), more synapses move to shallower weak meta-states, increasing the effective potentiation rate above the effective depression rate (Fig 2a). As the ratio of effective potentiation to depression rates increases, however, the fraction of synapses in weak meta-states decreases. Consequently, sensitivity to reward probability decreases as *p*_*r*_ becomes larger or smaller than 0.5.

**Fig 3:**
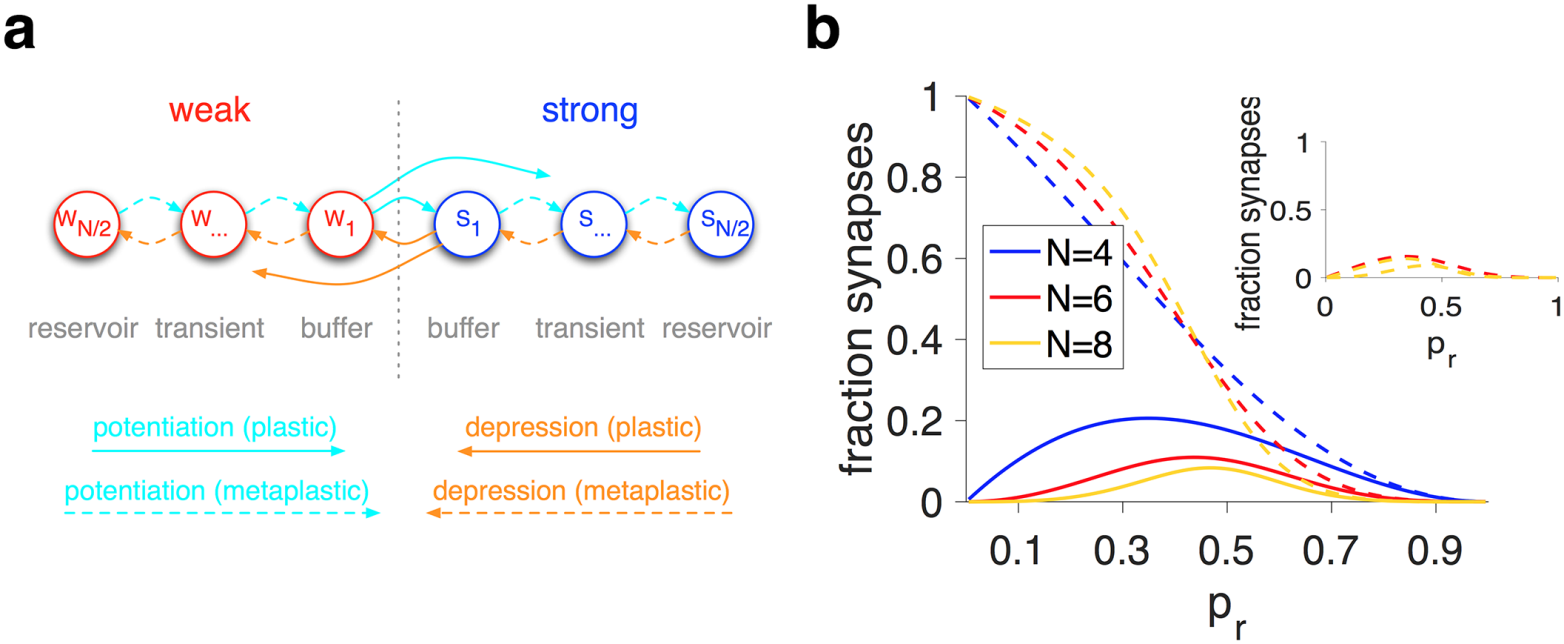
(**a**) Schematic of the reservoirs, buffers, and transient meta-states, and how synapses occupy these meta-states according to reward probability. (**b**) Plotted are the fractions of synapses in the weak reservoir (dashed lines) and buffer (solid lines) as a function of reward probability. The inset shows the fraction of transient meta-states for *N* = 6 and *N* = 8 models. Fractions in the strong reservoir, buffer, and transient meta-states are the mirror image (along *p*_*r*_ = 0.5) of their weak counterparts. As reward probability approaches 0.5, more synapses occupy transient and buffer meta-states, making the model more adaptable. As *p*_*r*_ deviates from 0.5, more synapses transition to reservoirs, enabling the model to protect the signal.

As noted above, in addition to the sensitivity-to-noise matching, adaptability of the superior models was optimized for a given level of noise. This optimization occurred because metaplasticity enabled superior models to form two separate sets of meta-states: reservoirs and buffers. Reservoirs, which are unique to metaplastic models, are the deepest sets of meta-states that cannot change their efficacy upon potentiation or depression events; they can only undergo metaplastic transitions (Fig 3a). Buffers, on the other hand, are the shallowest meta-states, and are able to undergo plastic transitions that change their synaptic efficacy. We refer to the remainder of the meta-states as ‘transient’. Because the superior models had reservoirs and buffers, they were able to keep a large proportion of their synapses in the weak or strong reservoirs (Fig 3b). Synapses within reservoirs were protected against changes in efficacy upon potentiation or depression events, and as a result, the signal could increase without increasing the level of noise.

**Fig 4:**
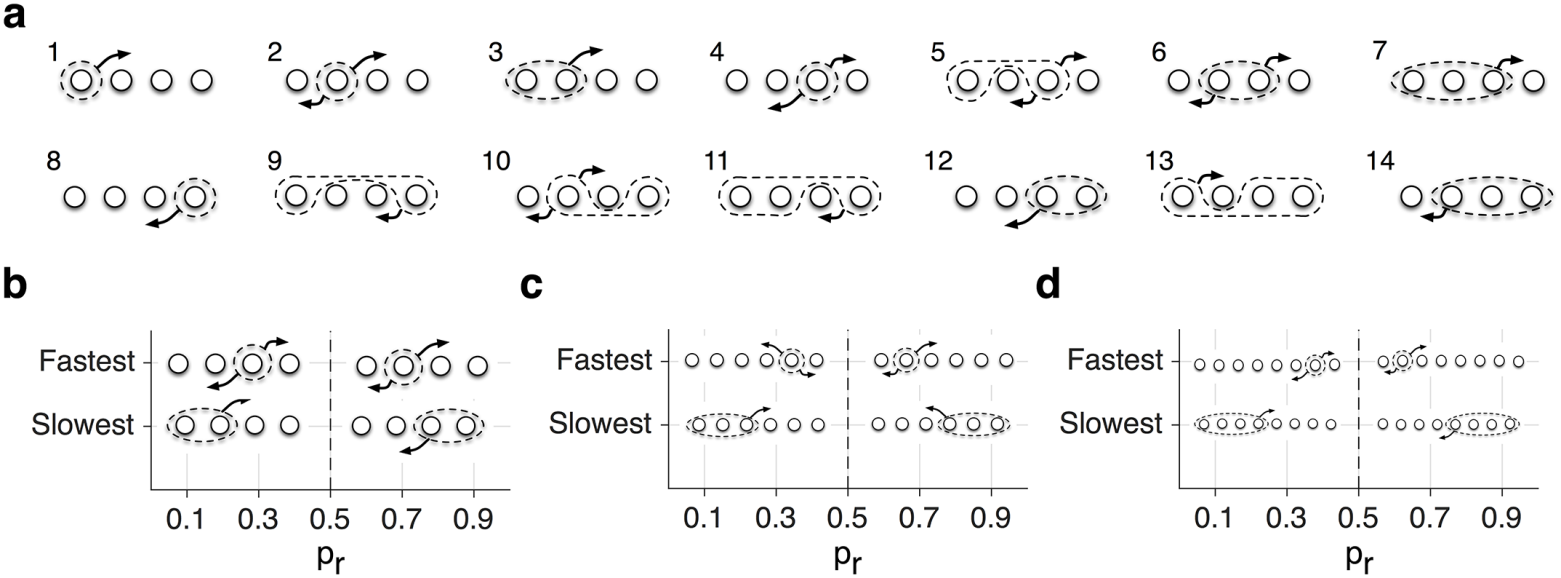
(**a**) Schematic of possible subsets of meta-states for *N* = 4 model. Overall, there are (2^*N*^ − 2) non-trivial subsets of meta-states for a given metaplastic model. (**b-d**) Plotted is a subset of meta-states with the fastest and slowest effective transition rates for different values of reward probability for the metaplastic models with *N* = 4,6,8. For superior models, the bottleneck (slowest) subset is always across the plastic boundary to minimize noise for a given level of adaptability, whereas the rapidly mixing (fastest) subsets consist of only transient meta-states in order to a build a quick connection between reservoir to buffer meta-states.

The adaptability in the model depends on the rates of transitions between all subsets of meta-states, whereas noise (in reward estimation) depends on the flow across the plastic boundary (i.e. transitions between weak and strong meta-states and vice versa). Therefore, to understand how the model’s adaptability is optimized for a given level of noise, we computed the ‘effective transition rate’ for all subsets of meta-states. The effective transition rate was defined as the outward flow of synapses out of that subset divided by the fraction of synapses in that subset. This quantity, which is closely related to the concept of conductance in Markov chains [18], measures how easily synapses could leave a subset of meta-states (Fig 4a; see Methods). Importantly, the model’s adaptability is constrained by its slowest effective transition rate.

In superior models, to reduce noise with a minimum cost to the adaptability, the slowest transition rates should be at the plastic boundary. We found that this was the case for all superior models (Fig 4). Interestingly, having the minimum effective transition rates at plastic transitions created a ‘bottleneck’ for the flow between weak and strong meta-states. This bottleneck helped reduce noise without significantly reducing the adaptability. The superior models with *N* > 4 also contained transient meta-states, with the fastest effective transition rates between buffers and reservoirs, resulting in improved adaptability (Fig 4c and 4d).

**Fig 5:**
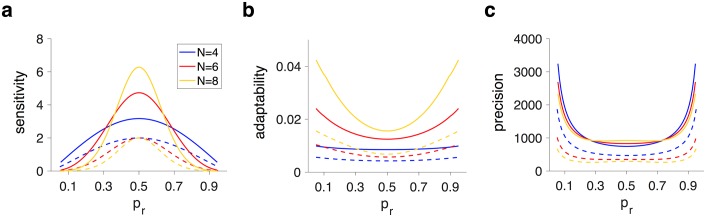
Comparisons between the behavior of the metaplastic models and equivalent binary plastic models with the effective learning rates for a given value of *p*_*r*_. (**a**) Plotted is sensitivity in three superior metaplastic models (solid curves) and their equivalent binary plastic models (dashed curves) as a function of *p*_*r*_. The equivalent plastic models are constructed using the effective learning rates for a given value of *p*_*r*_ and a metaplastic model. (**b-c**) Plotted is the adaptability and precision as a function of *p*_*r*_ for the same models presented in (a). The metaplastic models outperform equivalent binary plastic models in terms of sensitivity, precision, and adaptability for all values of reward probability.

This specific arrangement of meta-states and transitions between them, as well as the adjustment of the metaplastic model to reward probability, enabled metaplastic models to be more adaptable than corresponding binary plastic models. To demonstrate this superior adaptability, we used the effective learning rates for a given value of *p*_*r*_ to define an equivalent binary plastic model (*N* = 2 model) for any metaplastic model. We found that metaplastic models showed larger sensitivity to reward probability than equivalent plastic models (Fig 5). Moreover, metaplastic models were more adaptable and more precise than their equivalent plastic models. These results demonstrate that the dynamic adjustment of learning in metaplastic models is crucial for improving the APT, and that this adjustment cannot be achieved by simply replacing the learning rates in corresponding plastic models with the effective learning rates based on the superior metaplastic models.

### Superior family of metaplastic models for mitigating the APT

**Fig 6:**
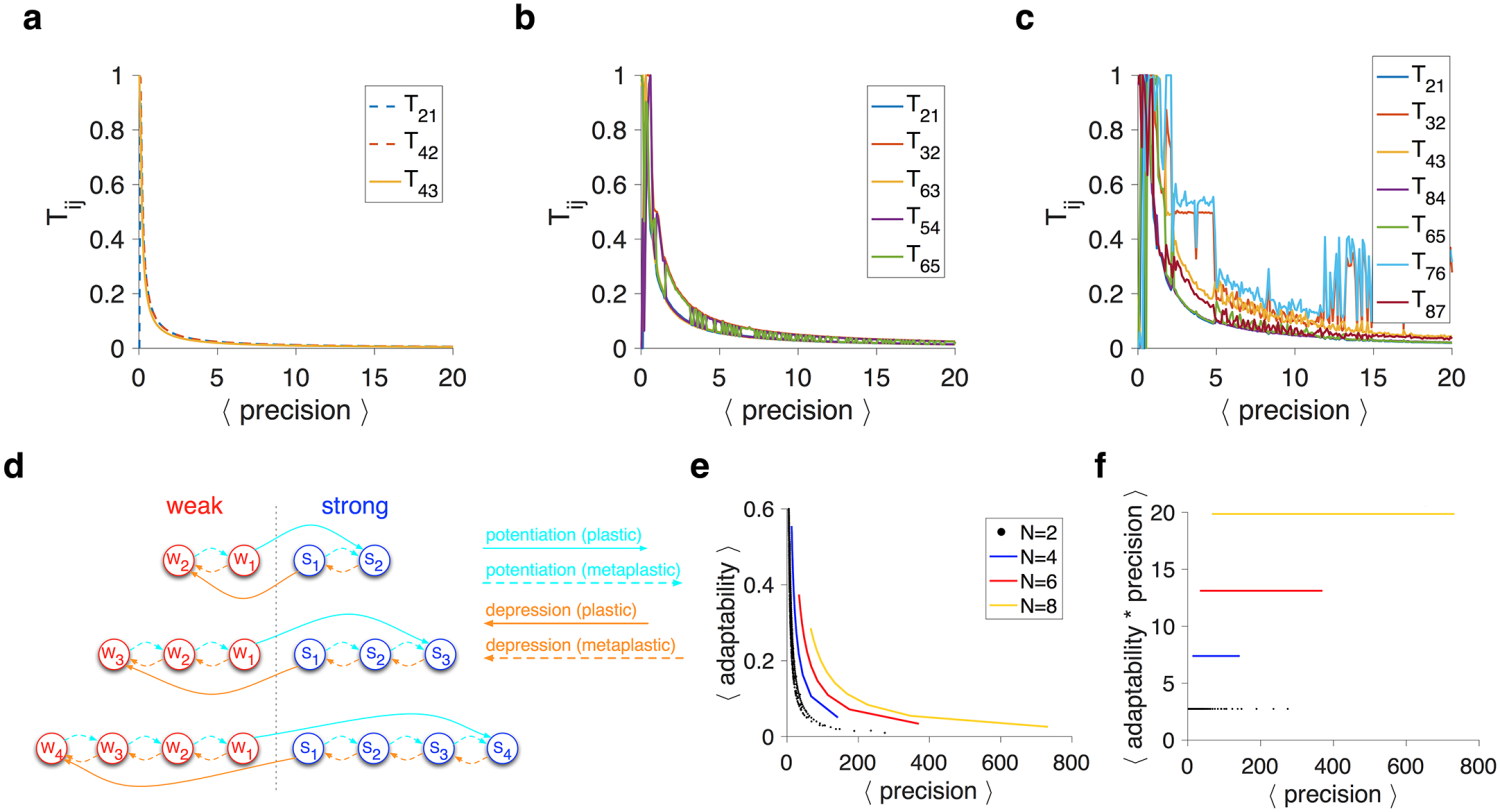
Transition probabilities in the superior metaplastic models and a superior family of metaplastic models with only one parameter. (**a-c**) Plotted are the transition probabilities for superior models with different numbers of meta-states (*N* = 4, 6, 8) for a given value of average precision. Only a few transition probabilities are non-zero, and the rest vary together, revealing the specific structure of metaplasticity that is useful for overcoming the APT. Finding optimal transition probabilities is more difficult for models with larger numbers of meta-states because the performance of those models are more robust against fluctuations in the model parameters. (**d**) The structure of the special family of metaplastic models with only one parameter (for *N* = 4,6,8 meta-states). (**e**) Adaptability as a function of the precision for the simple metaplastic models using the mean-field approach. (**f**) A  × ℙ as a function of the precision using the mean-field approach.

To further study the characteristics of superior metaplastic models, we next examined the transition probabilities in these models. We found that most transition probabilities were very close to zero, allowing for the creation of reservoirs and buffers, while non-zero transition probabilities varied proportionally to create models with different levels of adaptability and precision (Fig 6a–6c). For example, in metaplastic models with four meta-states (Fig 6a), three of six transition probabilities for potentiation were zero, two others were equal, and the last one was very close to those two other non-zero probabilities.

Based on these observations, we constructed a superior family of metaplastic models using a single parameter (transition probability). This was done to test whether such metaplastic models with only a single transition probability can significantly mitigate the APT. We found that even such simple metaplastic models can overcome the APT, and this ability was improved with additional meta-states (Fig 6d–6f). Overall, these results show that metaplastic models outperform plastic models, not because they have more parameters, but because they have a structure that allows for strong adjustment of learning.

These results illustrate that having more meta-states can improve the ability of metaplasticity to overcome the APT even for superior one-parameter models. The basic mechanism for this improvement is the existence of reservoirs, buffers, and a bottleneck for changing synaptic efficacy. Additional meta-states provide intermediate transitions between reservoirs and buffers that could increase signal and reduce noise without significantly decreasing the adaptability (Fig 6d–6f). As a result, models with larger numbers of intermediate meta-states show better matching of sensitivity to noise as well as more optimized adaptability for a given level of noise. Essentially, the specific structure for changing synaptic efficacy allows the models with a large number of meta-states to collect evidence (by transitioning synapses to shallower meta-states) before making a change.

### Validity of the mean-field approach

The results above were obtained using the mean-field (MF) approach. Although the MF approach could accurately estimate the signal, there are two components of the MF approach that could yield different results from the Monte Carlo (MC) simulations: adaptability and noise. As we show below, only the estimation of noise based on the MF approach is significantly different than noise based on the MC simulations. Nevertheless, our main findings based on MF also hold using MC simulations.

**Fig 7:**
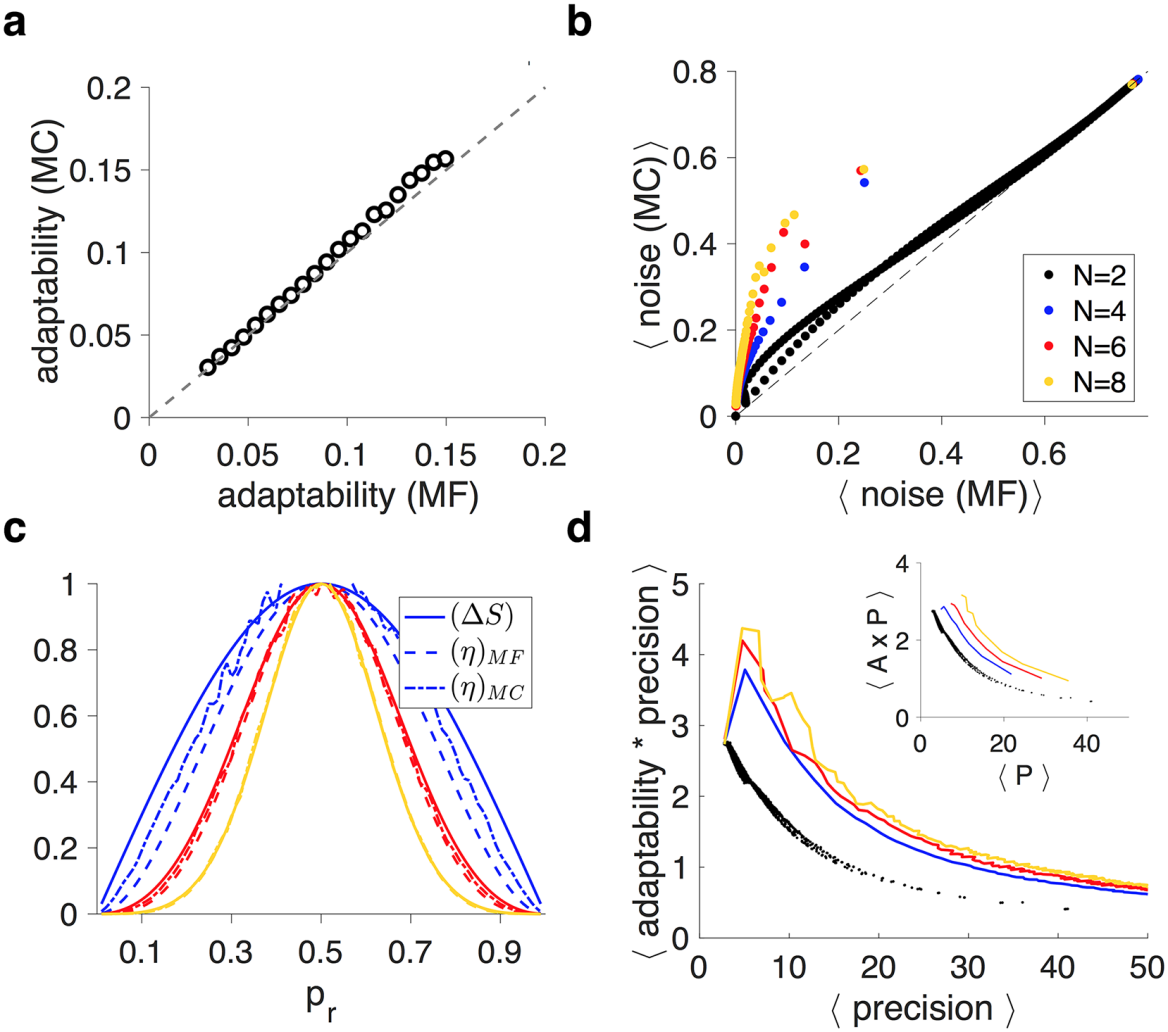
Comparison of results based on the mean-field approach and the Monte Carlo simulations. (**a**) Match between the adaptability calculated using the MF approach and the MC simulations for superior metaplastic models with four meta-states (*N* = 4). Adaptability in the MC simulations corresponds to the asymptomatic rate of convergence to the new equilibrium synaptic strength (i.e. signal). The convergence rate is computed over a jump in probability from 0.3 to 0.8 for different values of the transition probability in the model (from 0.05 to 0.25). The difference between adaptability computed with the two methods is very small. (**b**) Plotted is the noise computed using the MC simulations as a function of one-step noise for the binary plastic model and superior metaplastic models with different numbers of meta-states. One-step noise sets a lower bound for simulation noise. The MF approximation for noise becomes more accurate for higher adaptability. (**c**) Comparison of sensitivity-to-noise matching based on one-step noise and the MC simulations. Conventions are the same as in Fig 2c. (**d**) The APT in the binary plastic model and superior metaplastic models with different numbers of meta-states using the MC simulations. The inset shows the results for 1-parameter family using the MC simulations.

In the MF approach, adaptability is measured by the eigenvalue of the slowest decay mode of the transition matrix. However, what influences the estimation of reward probability is the synaptic strength (signal), since the synaptic efficacies of all weak meta-states or all strong meta-states are the same. That is, the asymptomatic rate of convergence to the new equilibrium or steady state of the synaptic strength could be different. Reaching steady state based on meta-states provides a lower bound for adaptability, since such equilibrium guarantees reaching steady state based on the synaptic strength but not vice versa. Comparing adaptability computed by the two methods, however, revealed only a small difference due to finite-size effects in the MC simulations (Fig 7a).

The only difference between the MF approach and MC simulations was the estimation of noise. Using the MF approach, the estimated noise was set to one-step noise, which is equal to the weighted average of changes in the steady state of synaptic strength due to a potentiation and depression event. The one-step noise converges to the actual noise if the adaptability is equal to 1. When the adaptability is different from 1, one-step noise underestimates the actual level of noise measured by real simulations (Fig 7b). Intuitively, this underestimation occurs because of the extra noise in the MC simulations due to fluctuation of the fractions of synapses in different meta-states around their steady-state values. While underestimation of noise in the MF approach increases with the number of meta-states, this effect is not strong enough to change the sensitivity-to-noise matching (Fig 7c). Moreover, the MC simulations showed the same order of models in their ability to overcome the APT (compare Figs 7d, 1d and 6d).

### Comparison with other models and metrics

In order to obtain the optimal structure of metaplasticity based on a general model, independently of a given task or set of task parameters, we measured adaptability as the rate at which the signal in the model approaches its steady state. Moreover, we measured precision as the ability of the model to differentiate between adjacent values of probability while considering noise. This “relative” definition of precision was adopted because any information stored at the synaptic level can be amplified (and thus be biased) by a change in the input firing rate.

Although superior metaplastic models are optimal in mitigating the APT based on the adopted definitions, a certain task or set of task parameters could favor certain models or certain model parameters. Alternatively, one could measure precision in an absolute fashion, for example as the difference between the estimated and actual reward probability. Therefore, we tested the performance of one-parameter superior models and a few competing models during a dynamic probability estimation task using both relative and absolute definitions of precision (see Methods). More specifically, we computed the performance of various models using the average difference between the transient signal and the steady state of the signal based on the actual value of reward probability at each time point in the task. We also computed the estimation error as the difference between the estimated and actual reward probability.

**Fig 8:**
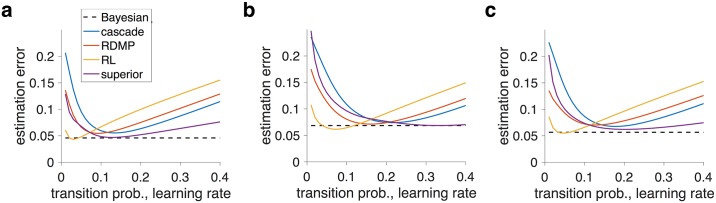
Comparison of the one-parameter superior models with competing models during a dynamic probability estimation task. Plotted are the average estimation errors as a function of the model parameter for the one-parameter superior model with six meta-states (*N* = 6), the heuristic RDMP model, the RL model with one learning rate, and the cascade model. The dotted black line shows the average estimation error for a hierarchical Bayesian model. Panels (a) and (b) show the results for a simple environment with *L* = 100 and 20, respectively. Panel (c) plots the performance for a complex environment with *L* between 10 and 100.

As expected, for a simple environment defined by the value *L* (the number of trials before reward probability is changed), the estimation error depends on the model parameter (transition probability or learning rate), except for when using the Bayesian model (Fig 8). For a large value of *L*, the RL model can achieve its optimal performance for a small value of learning rate, but the estimation error increases sharply for other values of the learning rate above that of the Bayesian model (Fig 8a). In contrast, the one-parameter superior family shows a small estimation error for a wide range of transition probability values. The cascade model shows larger estimation error since this model was designed to preserve its signal [19]. The performance of our previous heuristic metaplasticity model (RDMP) [3] falls between the superior and cascade models for larger values of the transition probability. Qualitatively, similar behavior was observed for performance in a simple environment with a smaller value of *L* (Fig 8b) and in a complex environment in which the value of *L* changes between blocks of trials (Fig 8c). Overall, these results illustrate that superior metaplastic models, which are identified by optimizing for the APT, can perform near-optimally over a wide range of parameter values during a dynamic estimation task.

In contrast, performance based on the absolute measure of estimation error (i.e. difference between the estimated and actual reward probability) revealed that one-parameter superior models perform worse than competing models (S3 Fig). This suboptimality of one-parameter superior models, however, stems from the steady-state signal (i.e. estimated reward probability) that strongly deviates from the actual reward probability (S4 Fig). This deviation, which allows the superior models to be very sensitive to changes in reward probability near 0.5, was much less pronounced in the cascade and RDMP models (signal in the RL model is equal to the actual reward probability). Overall, these results illustrate that superior metaplastic models, which are identified by optimizing for the APT, can perform close to optimally over a wide range of parameter values during a dynamic probability estimation task. However, these models can be suboptimal when an absolute metric is used for measuring precision (see Discussion).

### Robustness and importance of metaplasticity

**Fig 9:**
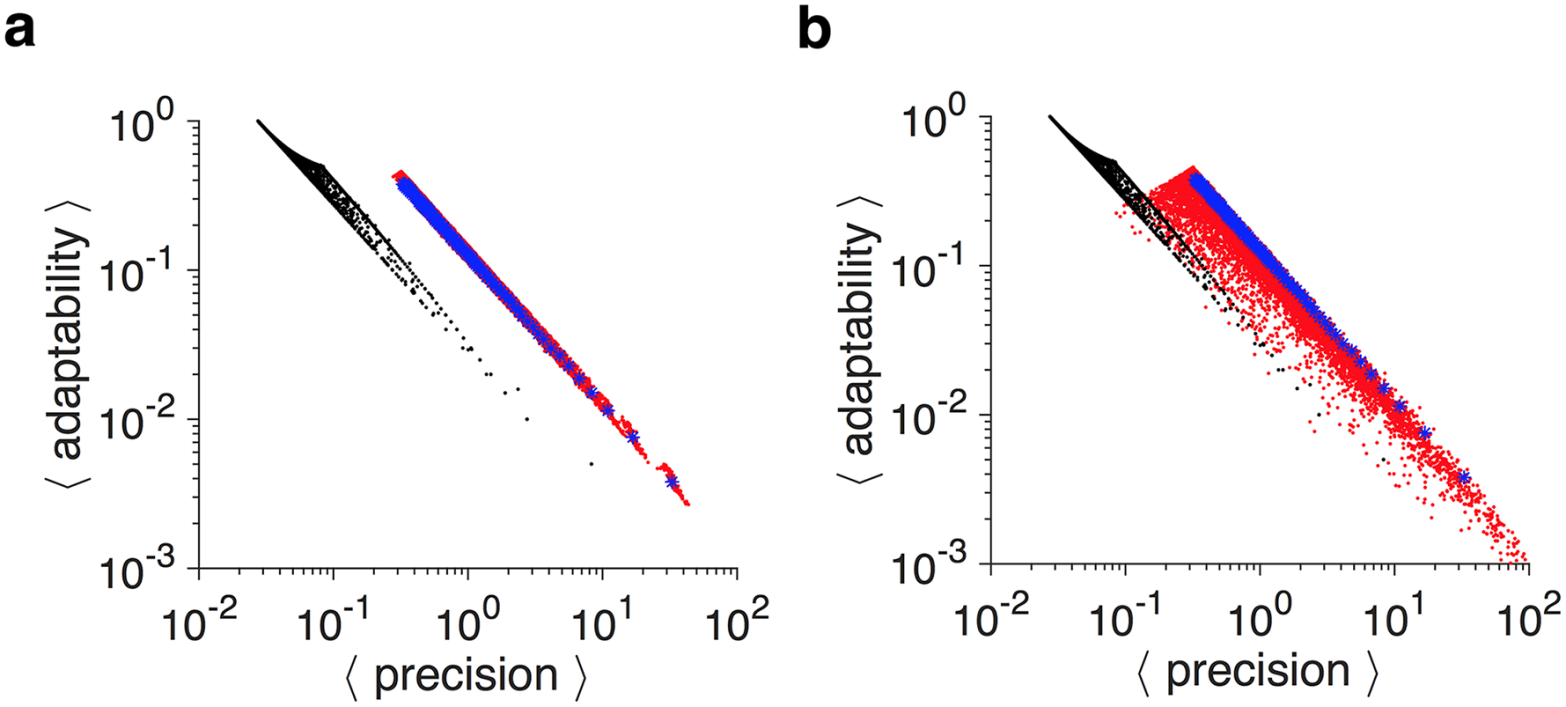
Robustness of metaplasticity to perturbations in model parameters. (**a**) Plotted is the average adaptability versus average precision in the superior models (blue dots) and superior models with six meta-states (*N* = 6) with perturbed transition matrices (red dots). The black dots show the binary plastic models for comparison (*N* = 2). To implement perturbation, the non-zeros elements in the potentiation and depression matrices of the superior models were independently perturbed by 10% of their original values (with Gaussian noise), with the constraint that the transition probabilities are positive. (**b**) The same as in (a) but with 50% perturbation.

In order to test the robustness of the metaplasticity solution, we examined how sensitive the superior solutions were with respect to changes in transition probabilities. To do so, we randomly perturbed the non-zeros elements in the potentiation and depression matrices of the superior models by a specific amount. We found that superior models show a high degree of robustness in their adaptability and precision against changes in their potentiation and depression transfer matrices as long as their transition topologies are not altered (i.e. the zero elements of transition matrices are kept zero) (Fig 9).

**Fig 10:**
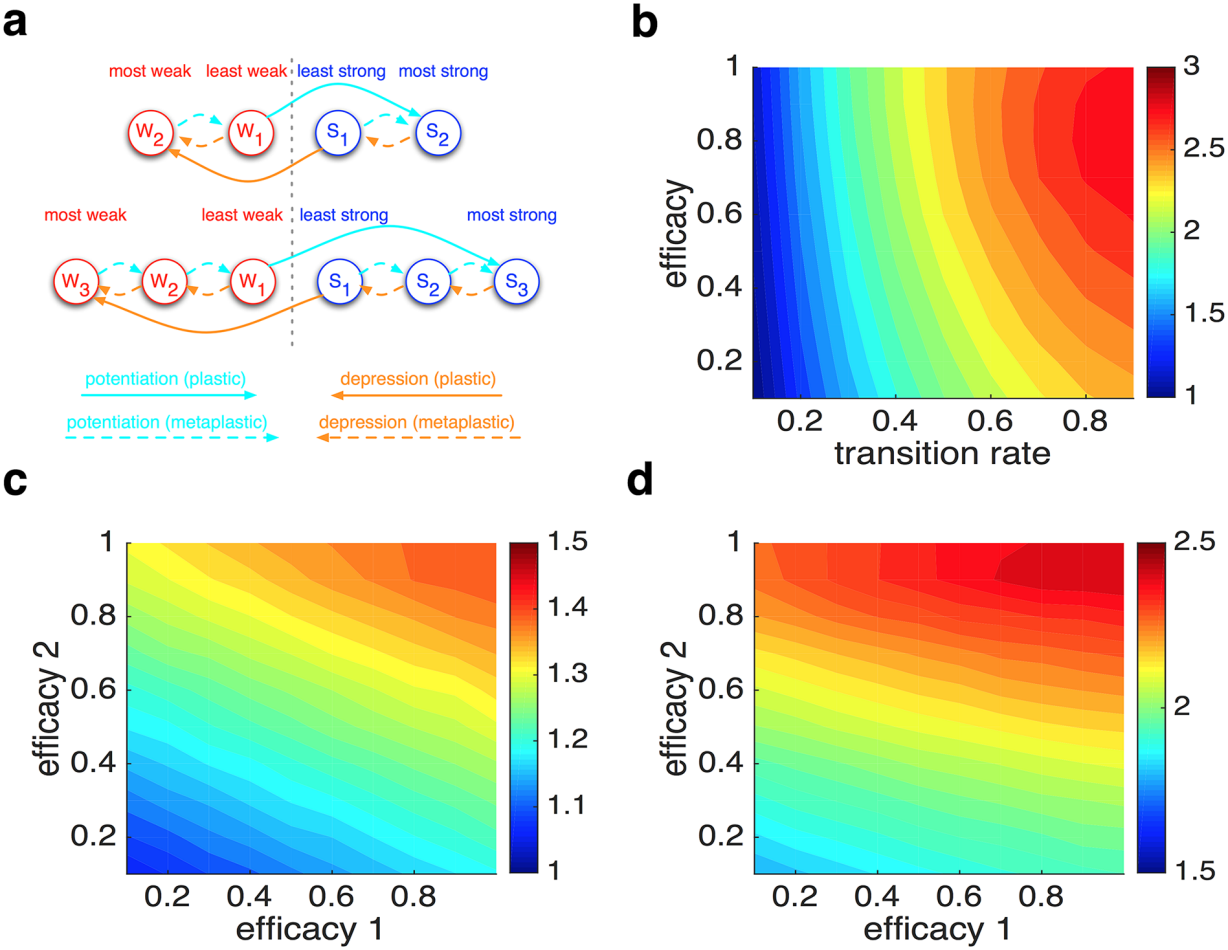
Graded plasticity reduces the ability to overcome the APT, indicating that metaplasticity is crucial for adaptive learning. (**a**) Schematic of the simple-family graded plastic models. This model has an equal number of states and transitions as the metaplastic model, but with different values of synaptic efficacy assigned to different states. (**b**) Plotted is the average A  × ℙ in the graded plastic models with four states as a function of the single transition rate and the efficacy of the least weak state (*efficacy* = 1 is equivalent to the *N* = 4 meta-plastic model). (**c**) Plotted is the average A  × ℙ in the graded plastic models with six states as a function of the efficacy of the weaker states when the single transition rate was set to 0.2 (*efficacy*_*1*_ = *efficacy*_*2*_ = 1 is equivalent to a superior meta-plastic model with six meta-states). (**d**) The same as in (c) but for the transition rate equal to 0.7. Overall, A  × ℙ monotonically increased as the graded plastic model became similar to the metaplastic model.

The ultimate test for whether metaplastic transitions are crucial for mitigating the APT is to replace these transitions with plastic ones (transitions that change synaptic efficacy) while keeping the same number of states and transitions. Therefore, we examined the APT in the simple family of metaplastic models, but with different values of synaptic efficacy assigned to different meta-states (Fig 10a; see Methods). This ‘graded’ plastic model could be reduced to the metaplastic model by setting equal values of synaptic efficacy for different weak or strong states. We found that A  × ℙ monotonically increased as the graded plastic model became more similar to the metaplastic model, reflecting the importance of metaplasticity to overcome the APT (Fig 10b–10d). Nevertheless, additional states in the plastic models improved the ability of these models to mitigate the APT beyond binary plastic synapses (S5 Fig) similar to improvement of memory storage capacity with more states [20]. Overall, these results demonstrate that metaplastic transitions are crucial for mitigating the APT.

## Discussion

The demands of learning in a changing world require a high degree of adaptability, which comes at the cost of low precision [4]. Here we show how metaplasticity, which is reflected in the unreliability of synaptic plasticity, can provide a solution for substantially overcoming the APT. More specifically, by optimizing the APT for a given level of precision, we identify crucial characteristics of superior metaplastic models. The superior models contain reservoir and buffer meta-states; synapses in reservoir meta-states do not change their efficacy upon reward feedback, whereas those in buffer meta-states can change their efficacy. Moreover, rapid changes in efficacy are limited to synapses occupying buffers, which provides a bottleneck that reduces noise without significantly decreasing adaptability. In contrast, more-populated reservoirs can generate a strong signal without manifesting any observable plasticity. The generation of reservoirs and buffers by metaplastic synapses results in the adjustments of learning, or the degree of plasticity, according to recent reward history. For example, when synapses occupy reservoir meta-states, which occurs with consecutive rewarded or unrewarded trials in a stable environment, the behavior should become less adaptable. However, when reward history changes over time, synapses mainly occupy buffer meta-states, causing more adaptable behavior. Overall, the model predicts that learning should be more sensitive to the reward sequence than what has previously been assumed (also see [3]).

Importantly, the results of one-parameter superior models and the MC simulations show that more meta-states can improve ability to overcome the APT and, in addition, give rise to more robust models for adaptive learning. These results are compatible with similar improvements in memory storage capacity with larger numbers of states [20]. Interestingly, the signal in the metaplastic models is a sigmoid-like function of reward probability. This illustrates that, for intermediate values of *p*_*r*_ (around 0.5), learning based on metaplastic synapses is more sensitive to changes in reward probability than learning based on plastic synapses. This sensitivity increases with the number of meta-states. Future experiments that can measure the sensitivity of probability learning can test this prediction of metaplasticity.

The basic mechanism for improvement with more meta-states is also related to the generation of reservoirs and buffers that create a bottleneck for changing synaptic efficacy, and not merely because of having a larger number of states as in graded synapses [21]. More specifically, additional meta-states provide intermediate transitions between reservoirs and buffers that could reduce noise without significantly compromising adaptability. Interestingly, it has been shown that in the framework of Markov chains, the eigenvalues and eigenvectors of models with bigger spectral gaps (i.e. more adaptable models) are less sensitive to perturbation of transition probabilities [22–25]. In other words, more adaptable models can produce signals without fine-tuning. Superior metaplastic models require only a few parameters, and their behavior is not very sensitive to these parameters.

As a higher-order form of plasticity, metaplasticity has been successfully used to explain paradoxical observations regarding synaptic plasticity by considering prior synaptic activity [12]. At the cognitive level, however, the computational power of metaplastic synapses has been mainly explored to address memory retention [19,26,27]. For example, Fusi and colleagues proposed the so-called cascade model to explain how memory could be protected from synaptic changes due to ongoing activity over large timescales [19]. By having multiple timescales associated with different meta-states, the cascade model can achieve high levels of both memory storage and retention time, and therefore, mitigate the ‘storage-retention’ tradeoff (i.e. a system which is good at storage would be poor at retention, and vice versa). A more recent study has shown that this tradeoff in memory systems can be further improved by having a large number of states that initially store memory quickly and then transfer memories to slower states [28]. This storage-retention tradeoff is exactly the opposite of the adaptability-precision tradeoff studied here, since memory systems are concerned with maintaining the signal whereas learning systems need to be adaptable. Nevertheless, it is encouraging that metaplasticity can mitigate two very different tradeoffs. Moreover, these results suggest that metaplasticity can be useful for estimating signals other than reward probability and is generalizable to other domains of learning for which adaptability and precision are both important.

Our results could also explain why plasticity protocols are unreliable and outcome plasticity is heterogeneous [29]. As we showed, superior metaplastic models create bottlenecks for changing synaptic efficacy, since such a property can reduce noise with minimal decrease in adaptability. However, limiting plastic transitions to those that occur from buffers would make many transitions invisible to measurement of change in synaptic efficacy. Therefore, until such a structure is specifically tested, plasticity protocols will be perceived as noisy and unreliable. Besides recent behavioral evidence [3], there is no direct electrophysiological evidence for the structure of metaplasticity proposed here. We hope that our study stimulates experimentalists to investigate this structure.

Using the difference between the estimated and actual reward probability as a measure of precision, we find that superior metaplastic models are suboptimal. This occurs because the signal in these models is biased to allow maximal sensitivity to reward probability for intermediate values of reward probability. However, if reward estimates have to be ultimately used for making choices as in binary decision-making tasks, it is more desirable to have a higher accuracy near such values of probabilities (0.5) where the outcome of the decision is more sensitive to the estimated value. Moreover, reward information has to be encoded and represented in the brain in a relative fashion in order to deal with a limited range of neural firing rates. Accordingly, we adopted a relative definition for precision that measures the ability to distinguish neighboring values of reward probability. Therefore, our results suggest that some of the suboptimality in the estimation of reward probability could be due to the biophysical limitations of the nervous system in encoding values.

Our proposal provides a new approach for studying synaptic plasticity and its contribution to brain computations. Our model predicts that a previous reward outcome (learning experience) not only contributes to learning and behavioral changes, but also affects subsequent induction of such changes within a specific time window. On the one hand, certain sequences of reward feedback cause the nervous system to become more receptive to subsequent similar feedback. On the other hand, consecutive feedback can shape future learning such that it is not responsive to feedback in the opposite direction. Understanding such propensity for and unresponsiveness to reward feedback could provide new insights into habit and addiction, respectively. Therefore, further investigations into metaplasticity, both at the behavioral and synaptic levels, could help researchers discover tools for improving learning, especially with respect to habits and addiction [30,31]. Overall, our work highlights an overlooked contribution of synaptic mechanisms to solving complex cognitive problems [32].

## Methods

### Metaplastic model

Our general model of metaplasticity consisted of multiple meta-states associated with two values of synaptic efficacy (weak and strong) and all possible transitions between these meta-states (Fig 1a). The metaplastic models have *N* distinct meta-states, half of which are associated with strong synaptic efficacy and half with weak. The model is completely specified with two transition matrices, one for a potentiation event ($T_{ij}^{+}$) and one for a depression event ($T_{ij}^{-}$) corresponding to rewarded and unrewarded trials, respectively. Here, we assumed that metaplastic transitions have a consistent order such that potentiation and depression events (on rewarded and unrewarded trials, respectively) create flows in opposite directions. This assumption also establishes weak and strong meta-states with different ‘depths’ such that deeper states are further from the plastic boundary (Fig 1a). Moreover, we assumed symmetry between information by reward and no-reward feedback, and thus only focused on mirror-symmetric flows. This assumption put another constraint on the potentiation and depression matrices:

$$T_{i,j}^{+}\;=T_{N-i,N-j}^{-}$$ (1)

Based on these assumptions, transition matrices for potentiation and depression events can be represented by lower-triangular and upper-triangular matrices:

$$T^{+}=\left[\begin{array}{cc} \begin{array}{cc} T_{11} & 0\\ T_{12} & T_{22} \end{array} & \begin{array}{cc} \cdots & 0\\ \cdots & 0 \end{array}\\ \begin{array}{cc} \vdots & \vdots \\ T_{1N} & T_{2N} \end{array} & \begin{array}{cc} \ddots & 0\\ \cdots & T_{NN} \end{array} \end{array}\right],\;\;\;\;\;T^{-}=\left[\begin{array}{cc} \begin{array}{cc} T_{NN} & \cdots \\ 0 & \ddots \end{array} & \begin{array}{cc} T_{2N} & T_{1N}\\ \vdots & \vdots \end{array}\\ \begin{array}{cc} \vdots & \cdots \\ 0 & \cdots \end{array} & \begin{array}{cc} T_{22} & T_{12}\\ 0 & T_{11} \end{array} \end{array}\right]$$ (2)

There are *N*(*N* − 1)/2 unique transition probabilities for models with *N* meta-states. The probability conservation was dictated by the transition flows out of any meta-state summing up to 1.

$$\sum_{j=1}^{N}T_{ij}=1,\;\;\forall i$$ (3)

### Mean-field approach

We assumed that the estimation of reward probability was performed by a set of synapses and, thus, reward probability was stored in the strength of these synapses. At any point in time, the signal (*S*) was defined as the difference between the fractions of synapses in the strong and weak meta-states,

$$S\left(t\right)\;=\;\Psi_{+}\left(t\right)\;-\;\Psi_{-}\left(t\right)$$ (4)

where $\Psi_{-}\;=\;\sum_{i=1}^{N/2}\Psi_{i}$ and $\Psi_{+}\;=\;\;\sum_{i=\frac{N}{2}+1}^{N}\Psi_{i}$ are fractions of synapses in the weak and strong meta-states, respectively. In the mean-field (MF) approximation approach, the average system dynamics is fully described by the average transition matrix for a given value of reward probability (${\bar{T}}_{ij}\;=\;p_{r}T_{ij}^{+}\;+\;\left(1\;-\;p_{r}\right)T_{ij}^{-}$). The eigenvector, *Ψ*, with an eigenvalue *λ* = 1 (the largest eigenvalue according to Perron-Frobenius theorem) of average transition matrix, ${\bar{T}}_{ij}$, provided the steady state of the model from which the average signal was calculated using Eq 4.

As a proxy for signal fluctuations around its average value, we introduced the concept of ‘one-step noise’ as the mean magnitude deviation from the average signal due to one potentiation or depression event:

$$\eta \;\equiv \;p_{r}\left|\langle S\rangle \;-\;S_{+}\right|\;+\;\left(1\;-\;p_{r}\right)\left|\langle S\rangle \;-\;S_{-}\right|$$ (5)

where 〈*S*〉 is the average signal based on the steady-state solution, and *S*_+_ and *S*_−_ are the signal values after the application of the potentiation or depression transition matrices on the steady-state solution, respectively. In general, noise at time (*t* + 1) is a combination of several components: (1) the attenuated transferred noise from the state of the system at time *t*; (2) the amount of noise generated in one step, from *t* to (*t* + 1); (3) the inherent noise involved in translating *p*(*t*) to a binary representation with potentiation and depression events; and finally (4) a finite size effect when dealing with a limited number of identical synapses. The one-step noise measures the second component and always underestimates the level of the noise in the model. The Monte Carlo simulations, however, contain the sum of the first three components mentioned above and thus capture the overall noise.

We defined precision as the ratio of the signal sensitivity and the one-step noise:

$$\mathbb{P}\;=\;\left(dS/dp_{r}\right)/\eta$$ (6)

Therefore, precision measures the discriminability between two adjacent reward probabilities based on their resulting signals. We chose this measure instead of the difference between the estimated and actual reward probability because the firing rate of neurons, which represents reward values, can be differentially scaled by their input firing rates. Therefore, the absolute difference may be irrelevant for the nervous system.

Finally, the adaptability of the model was defined as the rate of the decaying mode in the system, and was estimated using the difference between the second-largest eigenvalues (slowest decaying mode) of the average transition matrix and 1 ($A\;=\;1\;-\;\lambda_{2}$), also known as the spectral gap in the Markov chains literature. We chose this definition because it is not possible to reduce the dynamics of metaplasticity to arrive at one equation for the synaptic strength. As a result, adaptability measures the lower bound for the rate of convergence to the final steady state of the synaptic strength. Nevertheless, we found that our definition provides a good approximation for this rate (Fig 7a).

By focusing on the steady-state solution, the concept of learning rates in the binary plastic models (*N* = 2) can be generalized to higher *N* as the effective learning rates, ${\tilde{t}}^{\pm }$. The effective learning rates were defined as the relative change in the fraction of synapses in the weak or strong meta-states after a potentiation or depression event:

$${\left(T^{\pm }\;\Psi \right)}_{\pm }=\Psi_{\pm }+{\tilde{t}}^{\pm }\Psi_{\mp }$$ (7)

where (*T*^±^ Ψ)_±_ is the sum of the fraction of strong/weak meta-states. To examine transitions from a given subset of meta-states, we also defined the ‘effective transition rate’ as the outward flow of synapses from that subset, divided by the fraction of synapses in that subset (Fig 3a). The effective transition rate (${\tilde{T}}_{ab}$) assigns a single rate for outward transition from a set of meta-states *a* to a set of meta-states *b*. There are (2^*N*^ − 2) non-trivial ways that *N* meta-states can be partitioned into two disjoint, complementary subsets.

A closely related concept of conductance, *C*(*S*), for a given subset *S* in a Markov chain is defined as the outward flow from that subset divided by the minimum of occupancy in that subset, *π*(*S*), and occupancy in its complementary set *π*(*S*^*c*^). The magnitude of one-step noise is directly related to the effective transition rate when the two subsets are chosen based on their synaptic efficacy. The value of spectral gap (i.e. the difference between the second-largest eigenvalues of the average transition matrix and 1) is constrained by the minimum conductance among all possible subsets of meta-states [18].

### Monte Carlo simulations

The Monte Carlo simulations were performed by running multiple trials starting from a given initial state in environments with identical reward statistics (reward probability was the same but the reward sequence varied across different simulations). Data from an initial relaxation period was discarded to remove dependence on the initial state, and the relevant quantities were computed by averaging over the ensemble at a given time step or across time. Moreover, to further reduce the relaxation time, we started from the steady-state solution of the mean-field equation for the initial environment.

To measure the decay rate in the Monte Carlo simulations, we simulated the dynamics of signal in one-parameter superior models (with *N* = 4, and 6) in response to a sudden jump from reward probability of 0.3 to 0.8. We averaged over 100000 different instances of such simulations to obtain the asymptotic convergence of the signal. The asymptotic signal was then fit to an exponential function and the best fit for the time constant was computed using minimum squared error methods. We performed these simulations for different values of model parameters and transition probabilities between 0.05 and 0.25 (with 0.01 increments). The results of the Monte Carlo simulations were then compared with the models’ slowest mode using the mean-field approach.

### Finding the optimal solutions

The optimal solutions (i.e. upper-boundary in adaptability × precision vs. precision plot) were found in two stages. An initial upper envelope in the A × ℙ vs. ℙ (using discretization for ℙ) was constructed by random sampling of 10^7^ transition matrices. The transition matrices were divided into *n* bins according to their precision, ℙ, and the transition matrix with the highest value of A × ℙ in each bin was selected. These transition matrices were then used as the initial points for our optimization process. To avoid local minima, at the beginning of each iteration, a duplicated copy of the initial transition matrix with added small jitters was generated. All the resulting 2*n* transition matrices were used as the starting point of our optimization. At the end of each optimization iteration, the best solutions in each bin were selected out of all initial transition matrices, and the final outcome of our optimization procedure was used for the initial samples of the next iteration. For models with a large number of meta-states (*N* > 4), we conducted multiple iterations of the optimization process. The higher dimensional solutions are more robust against fluctuations, and optimized solutions can be found by increasing the bin numbers (initial points) and the number of optimization iterations. The optimization was constrained by keeping the sum of every column in transition matrices with positive elements to one. We used MATLAB’s ‘fminsearch’ function for the basic optimization process.

### Dynamic probability estimation task

To compare superior metaplastic models and a few competing models, we measured the performance of these models in a dynamic probability estimation task. In this task, the reward is provided on each trial based on a fixed probability. This probability, however, increases or decreases (with equal probability) by 0.1 every *L* number of trials, resulting in 11 different values of reward probability ([0, 0.1, 0.2, 0.3, 0.4, 0.5, 0.6, 0.7, 0.8, 0.9, 1.0]). Therefore, parameter *L* defines the level of volatility in this task. We simulated the behavior of various models in simple environments (environments with a fixed value of *L*) and in a complex environment where *L* can also change. For the complex environment, after each change in the reward probability, the value of *L* was selected randomly from the following set of values: [10, 20, 30, 40, 50, 60, 70, 80, 90, 100], subject to the constraint that overall lengths of blocks with a given value of *L* are similar.

We used two methods to compare the performance of various models (see below) in this task in terms of estimation error. In the first method, we computed the average difference between the transient signal and the steady state of the signal based on the actual value of reward probability at each time point during the task (relative estimation error). This was done because the signal (or the estimate of reward probability) in each model is relative, and there is a one-to-one mapping between the signal in a given model and the actual reward probability. In the second method, we computed the estimation error by the absolute value of the difference between the estimated and actual reward probability at each time point (absolute estimation error).

### Alternative models

In order to compare the performance of our model with competing models, we simulated four models and measured their performances in a dynamic probability estimation task. The first model was an RL model based on reward prediction error (see S1 Text). This model is equivalent to the binary plastic model (*N* = 2) and can be quantified with one or two parameters (learning rates). The second model was the so-called cascade model of Fusi et al. (2005)[19]. This cascade model also assumes metaplasticity and order for transitions between different meta-states similarly to our superior models. However, the cascade model has a different structure for transition than our simple family model and, moreover, transition probabilities become smaller for deeper meta-states. The third model was a heuristic model of reward-dependent metaplasticity (RDMP), which we have proposed to capture behavioral data during a dynamic learning and decision-making task [3]. Finally, we also simulated a hierarchical Bayesian model that directly estimates volatility and change in volatility in order to determine the amount of update based on reward feedback [1].

## Supporting information

S1 Text Equivalence of the RL model to the model with plastic synapses. (DOCX)

S2 Text Adaptability-precision tradeoff in the model with plastic synapses. (DOCX)

S1 Fig The APT in the binary plastic model (*N* = 2). Characteristics of the binary plastic model measured using different quantities as a function of reward probability for two sets of learning rates (*t*^+^ = 2 × *t*^−^ = 0.4 and *t*^+^ = 2 × *t*^−^ = 0.3). Adopting different learning rates improves the adaptability for certain values of *p*_*r*_ and improves precision for complementary values of *p*_*r*_, resulting in a strict tradeoff between adaptability and precision. A simple RL model based on RPE behaves similarly to the binary plastic model shown here.
(TIFF)

S2 Fig Example traces of the signal in response to a sudden change in reward probability in the binary plastic model (a, e), and three superior metaplastic models with different numbers of meta-states (*N* = 4, 6, and 8 in b-d and f-h, respectively). In each plot, the blue trace is an example estimate based on the shown reward sequence (tick marks on the top and bottom correspond to rewarded and unrewarded trials, respectively). Reward probability changed from 0.3 to 0.8 on trial 20. The black curve shows the average signal, and the green shade shows the signal plus/minus its s.e.m. Models in (a-d) are more adaptable, whereas models in (e-h) are more precise. For these simulations, example models were selected to have the same average precision. Overall, metaplastic models can improve adaptability without increasing noise in the signal (thinner green lines).
(TIFF)

S3 Fig Comparison of the performance of one-parameter superior models with competing models during a dynamic probability estimation task. Plotted are the absolute estimation errors as a function of the model parameter for the one-parameter superior model (*N* = 6), the heuristic RDMP model, the RL model with one learning rate, and the cascade model. The dotted black line shows the average estimation error for a hierarchical Bayesian model. Panels (a) and (b) show the results for a simple environment with *L* = 100 and 20, respectively. Panel (c) plots the performance for a complex environment with *L* between 10 and 100.
(TIFF)

S4 Fig Relationship between signal and actual reward probability in different models. Plotted are the steady state of signals in the superior metaplastic models (a), the cascade model (b), and the heuristic RDMP model (c) as a function of the actual reward probability, *p*_*r*_, for *N* = 6 number of meta-states and different transition probability and model parameters. Blue, red and golden curves correspond to transition probabilities equal to 0.1, 0.2, and 0.4, respectively. The superior metaplastic model deviates the most from the actual reward probability. Note that the signal in superior metaplastic model is independent of transition probability.
(TIFF)

S5 Fig The effect of graded synaptic efficacy on the ability to mitigate the APT. Plotted is the average APT as a function of the average precision using the Monte Carlo simulations in a family of graded plastic models with the same architecture as the one-parameter superior models. Panels (a) and (b) correspond to models with *N* = 4 and *N* = 6 graded states, respectively. The single transition probability is set to 0.1, 0.3, 0.5, 0.7, or 0.9 as indicated in the legend. The graded synaptic efficacies for different synaptic states for *N* = 4 models (a) were set to [-1, -*w*_*1*_, *w*_*1*_, 1]. Points with the same color in (a) correspond to values of *w*_*1*_ between 0.1 and 1 with 0.1 increments. The star represents *w*_*1*_ = 1 and corresponds to the superior metaplastic model. The graded synaptic efficacies for *N* = 6 models (b) were set to [-1, -*w*_*1*_, -*w*_*2*_, *w*_*2*_, *w*_*1*_, 1]. Points with the same color in (b) correspond to values of *w*_*1*_ and *w*_*2*_ between 0.1 and 1 with 0.1 increments. The star represents *w*_*1*_ = *w*_*2*_ = 1 and corresponds to the superior metaplastic model. The black dots are the RL model with different values of the learning rate.
(TIFF)
