# Supplementary material for: Optimal structure of metaplasticity for adaptive learning
Source: PLoS Comput Biol. 2017 Jun 28;13(6):e1005630. doi: 10.1371/journal.pcbi.1005630 (PMC5509349; doi:10.1371/journal.pcbi.1005630)
Supplement: S1 Text — (DOCX) [file pcbi.1005630.s001.docx]

**S1 Text. Equivalence of the RL model to the model with plastic synapses.** Here, we show that stochastic synapses without metaplasticity are equivalent to the RL model based on reward prediction error (RPE). A standard RL model based on reward prediction error (the difference between expected and actual outcomes) and with equal learning rates for rewarded and unrewarded trials can estimate reward probability (Sutton and Barto, 1998). This RL model is fully described by its value function, *V*. Its temporal dynamics in response to a feedback sequence is governed by a learning rate *δ* and the reward prediction error, which is the difference between expected and actual reward.

$$V \to V + \delta\times RPE$$

where $RPE = (1-V)$ or $(-V )$ on rewarded or unrewarded trials, respectively. The learning rates could differ on rewarded and unrewarded trials ($\delta_{+}$ and $\delta_{-}$) resulting in the following update rules:

$V \to V + \delta_{+} \times(1 - V )$on rewarded trials (Eq. S1)

$V \to V - \delta_{-} \times V$ on unrewarded trials

The average of *V* approaches reward probability *p_r_* in the environment when $\delta_{+}=\delta_{-}$.

A model with binary synapses (‘weak’ and ’strong’ states) that undergo stochastic reward-dependent plasticity can also provide an unbiased estimate of reward probability (Soltani and Wang, 2006; Soltani et al., 2006). In this model, weak synapses can be potentiated on rewarded trials with a probability *t*^+^ (potentiation rate), whereas strong synapses can be depressed on unrewarded trials with a probability *t*^−^ (depression rate). Therefore, the fraction of synapses in the strong state, Ψ_+_, is updated as the following:

$\Psi_{+} \to\Psi_{+} + t^{+}(1 - \Psi_{+})$ on rewarded trials (Eq. S2)

$\Psi_{+} \to\Psi_{+}- t^{-} \Psi_{+}$ on unrewarded trials

The equivalence between the RL and binary plastic models ($N = 2$) can be seen by comparing the strong-state occupancy percentage with the value function and replacing $\delta_{\pm}$ with $\tilde{t}^{\pm}$ in Equations S1 and S2.

**References**

Soltani, A., Lee, D., and Wang, X.-J. (2006). Neural Mechanism for Stochastic Behavior During a Competitive Game. Neural Networks, 19, 1075–1090.

Soltani, A., and Wang, X.-J. (2006). A biophysically based neural model of matching law behavior: melioration by stochastic synapses. The Journal of Neuroscience, 26(14), 3731–3744.

Sutton, R.S. and Barto, A.G. (1998) Reinforcement Learning: An Introduction, MIT Press, Cambridge, MA
