# Supplementary material for: Optimal structure of metaplasticity for adaptive learning
Source: PLoS Comput Biol. 2017 Jun 28;13(6):e1005630. doi: 10.1371/journal.pcbi.1005630 (PMC5509349; doi:10.1371/journal.pcbi.1005630)
Supplement: S2 Text — (DOCX) [file pcbi.1005630.s002.docx]

**S2 Text. Adaptability-precision tradeoff in the model with plastic synapses.** In the model with plastic synapses ($N = 2$), we defined the signal as the difference between the fractions of synapses in the strong and weak states, $S = \Psi_{+}- \Psi_{-}$. For the value of reward probability equal to $p_{r}$, the average signal is equal to

$\left\langle S \right\rangle=\frac{p_{r}t^{+}-(1-p_{r})t^{-}}{p_{r}t^{+}+(1-p_{r})t^{-}}$ (Eq. S3)

Therefore, the signal ‘sensitivity,’ defined as the derivative of the average signal with respect to $p_{r}$, is equal to

$\frac{d\left\langle S \right\rangle}{dp_{r}}=\frac{2t^{+}t^{-}}{\left[ p_{r}t^{+}+(1-p_{r})t^{-} \right]^{2}}$ (Eq. S4)

The ‘one-step noise’, defined as the mean magnitude deviation from the average signal in one time step, is equal to

$\eta\equiv p_{r}\left| \left\langle S \right\rangle-S_{+} \right|+\left( 1 - p_{r} \right)\left| \left\langle S \right\rangle-S_{-} \right|=\frac{4p_{r}\left( 1 - p_{r} \right)t^{+}t^{-}}{p_{r}t^{+}+(1-p_{r})t^{-}}$ (Eq. S5)

Hence, the precision is equal to

$\mathbb{P\equiv}\frac{d\left\langle S \right\rangle/dp_{r}}{\eta}=\frac{1}{2p_{r}\left( 1 - p_{r} \right)\left[ p_{r}t^{+}+(1-p_{r})t^{-} \right]}$ (Eq. S6)

The adaptability is defined as the rate of decaying mode in the system. In $N = 2$ models, the rate of approach toward the average signal is fully governed by the weighted average of the learning rates:

$\mathbb{A}= p_{r}t^{+} + (1 - p_{r})t^{-}$ (Eq. S7)

Finally, $N = 2$ models show a strict APT since the product of adaptability and precision is independent of model parameters and only depends on reward probability:

$\mathbb{A \times P=}\frac{1}{2p_{r}(1-p_{r})}$ (Eq. S8)

In general, adaptability and noise are related to each other in the model with plastic synapses and in the RL model. This is because an increase in transition probabilities between weak and strong states causes larger flows between the two states, which in turn increases noise. Importantly, adopting different transition probabilities (or learning rates in RL) for potentiation and depression events cannot improve the APT. Rather, it affects the average values for adaptability and precision individually (Fig. 1d and S1 Fig).
