# Supplementary figures and images for: Optimal structure of metaplasticity for adaptive learning
Source: PLoS Comput Biol. 2017 Jun 28;13(6):e1005630. doi: 10.1371/journal.pcbi.1005630 (PMC5509349; doi:10.1371/journal.pcbi.1005630)

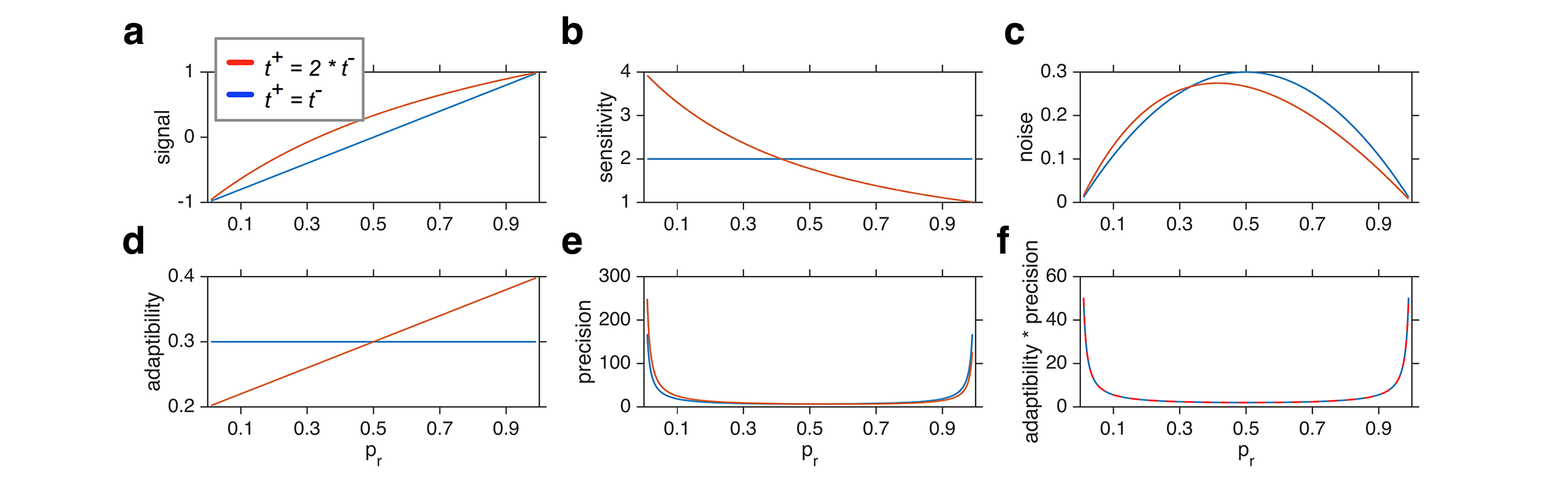

Supplement: S1 Fig — Characteristics of the binary plastic model measured using different quantities as a function of reward probability for two sets of learning rates (t+ = 2 × t− = 0.4 and t+ = 2 × t− = 0.3). Adopting different learning rates improves the adaptability for certain values of pr and improves precision for complementary values of pr, resulting in a strict tradeoff between adaptability and precision. A simple RL model based on RPE behaves similarly to the binary plastic model shown here. (TIFF) [file pcbi.1005630.s003.tiff]

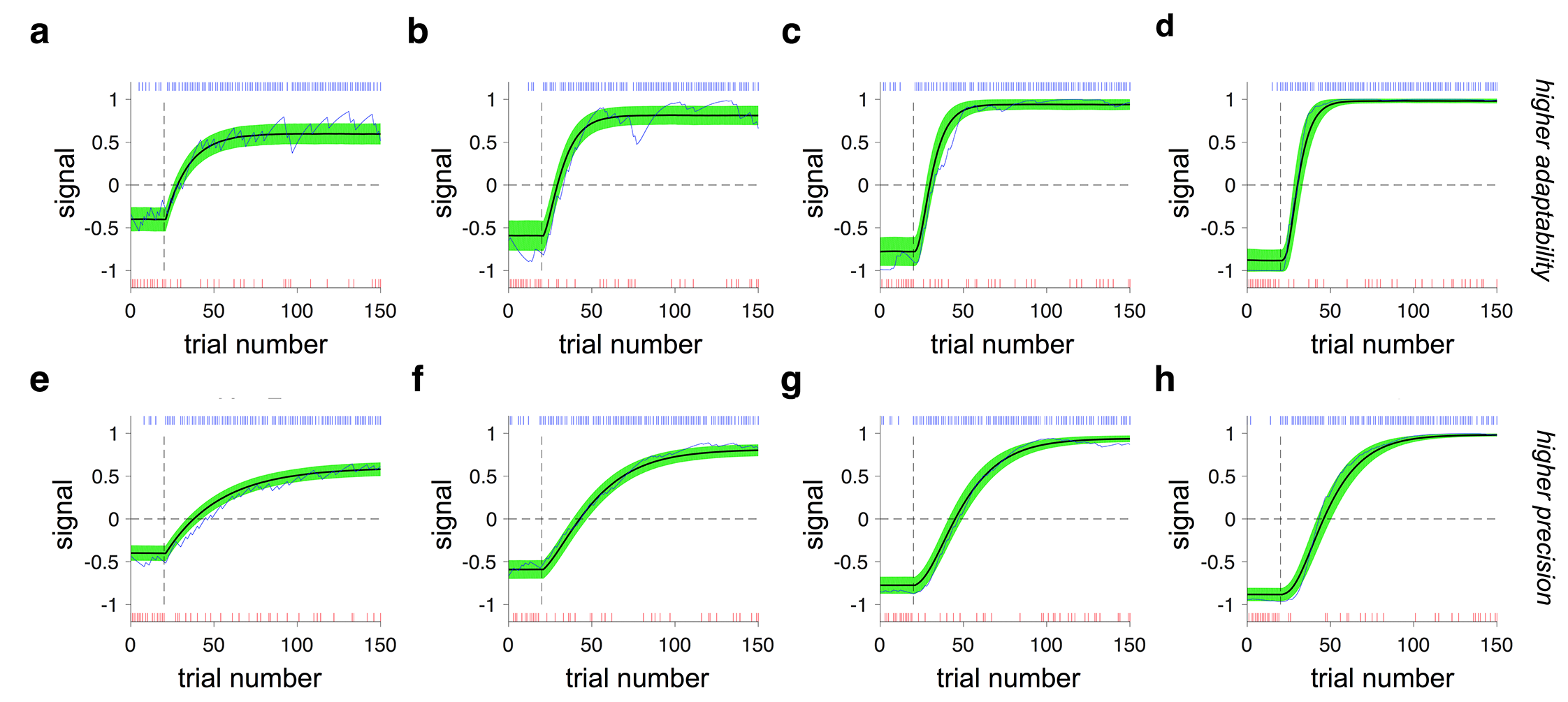

Supplement: S2 Fig — In each plot, the blue trace is an example estimate based on the shown reward sequence (tick marks on the top and bottom correspond to rewarded and unrewarded trials, respectively). Reward probability changed from 0.3 to 0.8 on trial 20. The black curve shows the average signal, and the green shade shows the signal plus/minus its s.e.m. Models in (a-d) are more adaptable, whereas models in (e-h) are more precise. For these simulations, example models were selected to have the same average precision. Overall, metaplastic models can improve adaptability without increasing noise in the signal (thinner green lines). (TIFF) [file pcbi.1005630.s004.tiff]

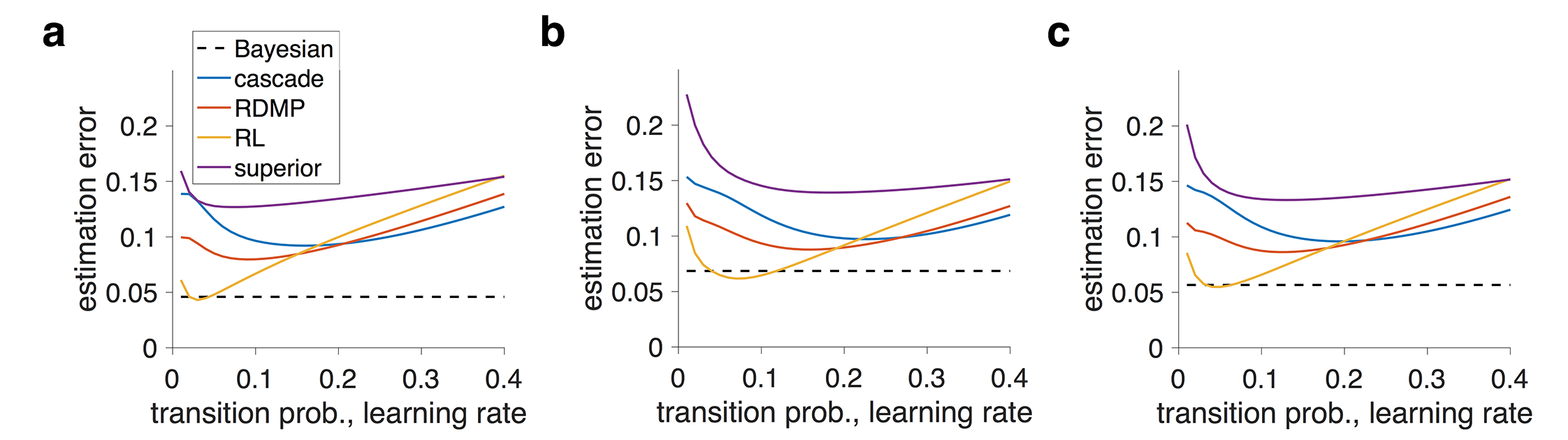

Supplement: S3 Fig — Plotted are the absolute estimation errors as a function of the model parameter for the one-parameter superior model (N = 6), the heuristic RDMP model, the RL model with one learning rate, and the cascade model. The dotted black line shows the average estimation error for a hierarchical Bayesian model. Panels (a) and (b) show the results for a simple environment with L = 100 and 20, respectively. Panel (c) plots the performance for a complex environment with L between 10 and 100. (TIFF) [file pcbi.1005630.s005.tiff]

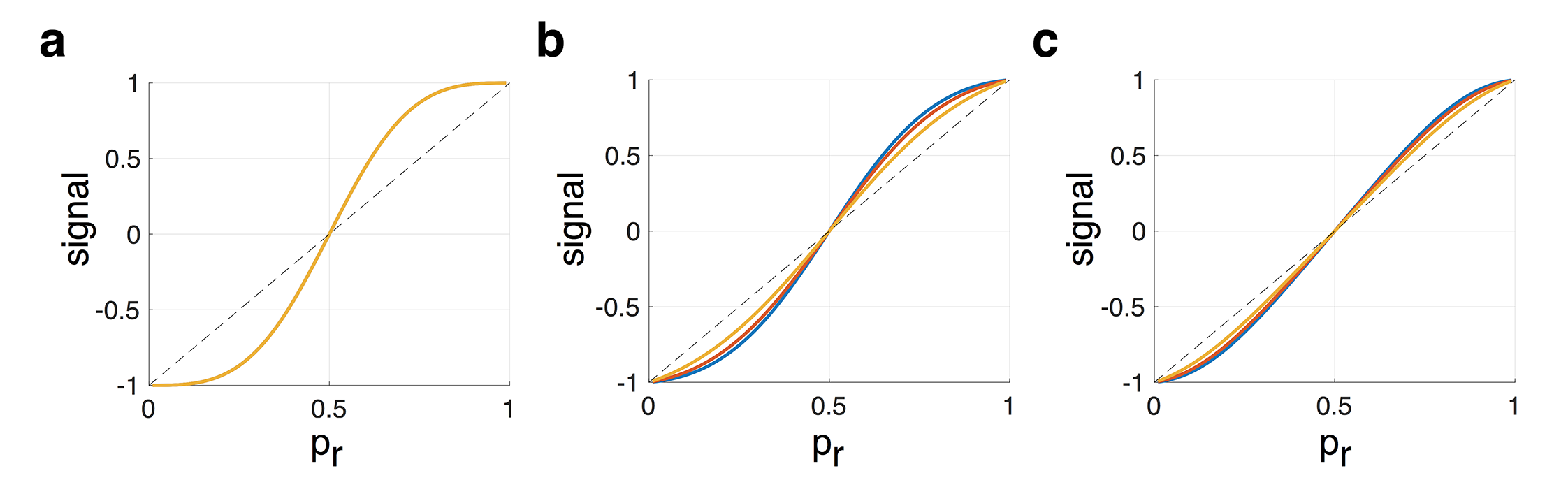

Supplement: S4 Fig — Plotted are the steady state of signals in the superior metaplastic models (a), the cascade model (b), and the heuristic RDMP model (c) as a function of the actual reward probability, pr, for N = 6 number of meta-states and different transition probability and model parameters. Blue, red and golden curves correspond to transition probabilities equal to 0.1, 0.2, and 0.4, respectively. The superior metaplastic model deviates the most from the actual reward probability. Note that the signal in superior metaplastic model is independent of transition probability. (TIFF) [file pcbi.1005630.s006.tiff]

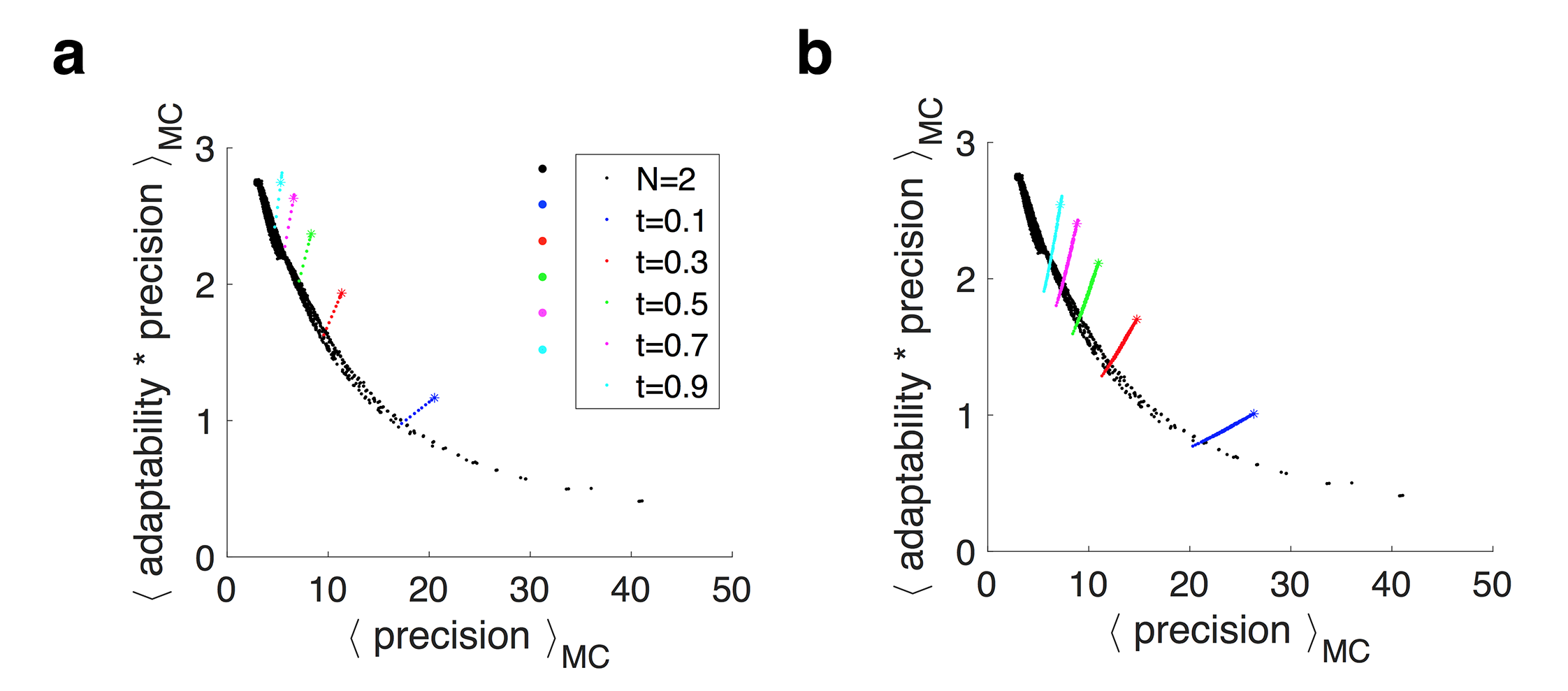

Supplement: S5 Fig — Plotted is the average APT as a function of the average precision using the Monte Carlo simulations in a family of graded plastic models with the same architecture as the one-parameter superior models. Panels (a) and (b) correspond to models with N = 4 and N = 6 graded states, respectively. The single transition probability is set to 0.1, 0.3, 0.5, 0.7, or 0.9 as indicated in the legend. The graded synaptic efficacies for different synaptic states for N = 4 models (a) were set to [-1, -w1, w1, 1]. Points with the same color in (a) correspond to values of w1 between 0.1 and 1 with 0.1 increments. The star represents w1 = 1 and corresponds to the superior metaplastic model. The graded synaptic efficacies for N = 6 models (b) were set to [-1, -w1, -w2, w2, w1, 1]. Points with the same color in (b) correspond to values of w1 and w2 between 0.1 and 1 with 0.1 increments. The star represents w1 = w2 = 1 and corresponds to the superior metaplastic model. The black dots are the RL model with different values of the learning rate. (TIFF) [file pcbi.1005630.s007.tiff]
